# Reduced Kidney Function, Albuminuria, and Risks for All-cause and Cardiovascular Mortality in China: A Population-based Cohort Study

**DOI:** 10.1186/s12882-017-0603-9

**Published:** 2017-06-07

**Authors:** Jinwei Wang, Fang Wang, Shiwei Liu, Maigeng Zhou, Luxia Zhang, Minghui Zhao

**Affiliations:** 1 Renal Division, Department of Medicine, Peking University First Hospital, 8 Xishiku Street, Xicheng District, Beijing, China; 2 Institute of Nephrology, Peking University, Beijing, China; 3 Key Laboratory of Renal Disease, National Health and Family Planning Commission of the People’s Republic of China, Beijing, China; 4 Key Laboratory of Chronic Kidney Disease Prevention and Treatment, Ministry of Education, Beijing, China; 5 National Center for Chronic and Non-communicable Disease Control and Prevention, Chinese Center for Disease Control and Prevention, Beijing, China

**Keywords:** Cardiovascular diseases, China, Chronic kidney disease, Glomerular filtration rate, Mortality, Urinary albumin-to-creatinine ratio

## Abstract

**Background:**

Previous studies have indicated that reduced kidney function and albuminuria are associated with increased risk of mortality and adverse cardiovascular outcomes, however, the evidence from the Asian population is limited. We investigated the association between the indicators of chronic kidney disease (CKD) and all-cause mortality, as well as cardiovascular mortality among a general population in China.

**Methods:**

We conducted an observational study among 47,204 Chinese adults, from a cross-sectional survey, whose survival status is identified through December 31, 2013. Estimated glomerular filtration rate (eGFR) and urinary albumin-to-creatinine ratio (ACR) were used as indicators of CKD. We determined the rates of all-cause and cardiovascular mortality.

**Results:**

The incidence rates for both all-cause and cardiovascular mortality increased with the advanced stages of reduced eGFR or elevated ACR. Elevated ACR levels were found to be significantly associated with increased risk of both all-cause and cardiovascular mortality, as shown in the Cox proportional hazards regression model. The multivariable adjusted hazard ratios (HR) associated with all-cause mortality were 1.26 (95% confidence interval [CI]: 1.04–1.53) for those with ACR 30–299 mg/g and 2.07 (95% CI: 1.40–3.04) with ACR ≥ 300 mg/g, compared to those with ACR <30 mg/g. The corresponding HRs for the above ACR levels associated with cardiovascular mortality were 1.08 (95% CI: 0.77–1.50) and 2.32 (95% CI: 1.31–4.12), respectively. We did not identify reduced eGFR as a risk predictor in the multivariable adjusted model for the adverse outcomes in the population, however, an interaction between eGFR and age were detected. Stratified analyses revealed that the associations of reduced eGFR (<60 mL/min/1.73 m^2^) with all-cause mortality were prominent among participants aged less than 65 years.

**Conclusions:**

Albuminuria was associated with an elevated risk of all-cause and cardiovascular mortality among the Chinese population, however, the association of reduced kidney function with all-cause mortality was not clear.

**Electronic supplementary material:**

The online version of this article (doi:10.1186/s12882-017-0603-9) contains supplementary material, which is available to authorized users.

## Background

Chronic kidney disease (CKD), defined by reduced glomerular filtration rate (GFR) or presence of markers for kidney damage, has rapidly emerged as a severe global health problem, with the population prevalence of CKD ranging from 10% to 16% in various regions [1–4]. Meanwhile, recent studies revealed that independent, strong, and graded associations were observed between clinical prognosis and two important markers of CKD: reduced GFR and increased urinary albumin excretion [5], suggesting the inclusion of albumin in CKD staging in order to improve risk stratification [6].

In China, the largest developing country, the prevalence of CKD is 10.8%, meaning 119.5 million adults have reduced estimated glomerular filtration rate (eGFR) and/or albuminuria [7]. Understanding the consequences of CKD based on relevant markers is important for making relevant decisions regarding prevention and treatment of CKD. However, most studies were from developed countries, and studies based on Asian general populations are limited. Previously, Wen et al. reported an inverse association between eGFR and risk of all-cause as well as cardiovascular disease (CVD) mortality, among patients receiving regular health check-ups in Taiwan. Positive urine proteinuria, which was determined by trace and higher levels in the dipstick test, was also found to be significantly associated with an increased risk of adverse outcomes [4]. Nonetheless, the high incidence of renal replacement therapy in Taiwan, and different etiology of end-stage renal disease in Taiwan compared with mainland China, limit generalizability of the study [8, 9].

To address the limitations of the existing studies, we have initiated the present study, on a nationally representative sample of the Chinese adult population, to evaluate the association between two markers of CKD based on a single measurement and mortality.

## Methods

### Study design and population

Our study is based on a cross-sectional study with baseline and follow-up information on participants to identify the adverse outcomes of interest. The study population was the participants of the China National Survey of Chronic Kidney Disease, who were general non-institutionalized adults (18 years or older) recruited from 13 provinces of China via a multistage, stratified sampling method [7]. All on-site screenings were done between January 2007 and December 2012. A total of 50,550 people were invited to participate, of whom 47,204 completed all essential investigations. The study was conducted in adherence to the Declaration of Helsinki and approved by the Institutional Review Board (IRB) committee of Peking University First Hospital. Written informed consent from participants was obtained prior to data collection.

### Measures of kidney damage and kidney function

Albumin creatinine ratio (ACR) and eGFR were evaluated in the study. Blood and urine samples were analyzed at the central laboratory in each province. A fresh morning spot urine sample or morning urine sample stored at 4 degree Celsius for less than 1 week was used to measure urinary albumin and creatinine with the methods of immune-turbidimetric testing and Jaffe’s kinetic method, respectively. ACR (mg/g creatinine) was the ratio of the two measurements. We categorized ACR into three groups of <30 mg/g, 30–299 mg/g and ≥300 mg/g, which corresponds to A1, A2, and A3 categories, respectively, suggested by the Kidney Disease Improving Global Outcomes (KDIGO) initiative. Serum creatinine was measured from overnight fasting blood collected by venipuncture, and urinary creatinine was measured from an overnight fasting urine sample. eGFR was calculated with the Chronic Kidney Disease Epidemiology Collaboration (CKD-EPI) equations for White or other (not Black) [10] race. We categorized eGFR into three groups of ≥90, 60–89, and <60 mL/min/1.73 m^2^. We also estimated eGFR by using an equation developed by a modified Modification of Diet in Renal Disease (MDRD) equation in a sensitivity analysis [11]. All study laboratories successfully completed a standardization and certification program by calibrating creatinine measurements with samples at the laboratory of Peking University First Hospital (Beijing, China), where the modified equation was developed.

### Outcomes

Data of the China National Survey of Chronic Kidney Disease were linked to the Master Death file (Jan 1, 2008-Dec 31, 2013) of the China Cause of Death Reporting System to determine all-cause mortality and cause-specific mortality [12]. The system is managed by the Chinese Center for Disease Control and Prevention and the death cases are reported by nearly all hospitals across China with death certificates and International Classification of Diseases (ICD) coded causes, through an internet-based reporting system. The under-reporting rate was estimated to be 16.68% during 2006–2008 [13]. Personal identification numbers were used as the key variable for linking the data, and information, including name, gender, birthdate, and home address, was used to verify the accuracy of linkage. Causes of death in ICD codes I00-I99 were classified as cardiovascular disease. We used the de-identified merged data for analyses.

### Covariates

All subjects completed a questionnaire documenting their socio-demographic status, personal and family health history, and lifestyle behaviors with the assistance of trained general practitioners and nurses. We used two levels of education in the analyses: junior high school or lower (9 years of education or less) and high school or higher (9 or more years of education). Current smoking was defined as smoking every day for at least one year. History of myocardial infarction (MI), and use of stroke and nephrotoxic medications (non-steroidal anti-inflammatory drugs and natural remedies and herbal preparations containing aristolochic acid) were also collected. Height and weight were measured according to the standard protocol. Body-mass index (BMI) was calculated as weight in kilograms divided by height in meters squared. Blood pressure (BP) was measured by sphygmomanometer, three times at 1 min intervals. The mean of the three readings was calculated. Hypertension was defined as a systolic BP ≥140 mmHg, or diastolic BP ≥90 mmHg, or self-reported use of antihypertensive medications in the last 2 weeks, or any self-reported history of hypertension. Fasting blood glucose was measured enzymatically with a glucose oxidase method. Diabetes was defined as fasting plasma glucose ≥126 mg/dL (7.0 mmol/L), or by the use of hypoglycemic agents, or any self-reported history of diabetes. Serum triglycerides (TG) and low density lipoprotein cholesterol (LDL-C) were measured using an enzymatic method with commercially available reagents (Jiancheng Bioengineering Institute, Nanjing, China).

### Statistical analysis

Continuous data were presented as mean ± standard deviation (SD), except for ACR and TG, which were presented as median (interquartile range [IQR]) because of large skewness. Categorical variables were presented as number and proportion. The relevant characteristics were described in total population, eGFR < or ≥60 mL/min per 1.73m^2^, and ACR ≥ or <30 mg/g. The follow-up time for each participant in the study was the length of time between the date of on-site examination and the end of follow-up (Dec 31, 2013) or date of death, whichever came first. All-cause and cardiovascular specific mortality rates were calculated as the number of death per 1000 person-years. We depicted the cumulative survival rates for all-cause and cardiovascular mortality according to categories of CKD indicators and compared them by using a log-rank test. We did not account for the sampling weight in the analysis, because the purpose of the current study was to evaluate the effect of CKD indicators rather than to report a nationally representative prevalence rate.

We used Cox proportional-hazards models to evaluate the effect of the CKD indicators on mortality, using eGFR ≥ 90 mL/min per 1.73m^2^ or ACR < 30 mg/g as reference. Univariable and multivariable adjusted hazard ratios (HRs) and 95% confidence intervals(CI) were reported. Covariates included in the multivariable adjusted regression model were age (a continuous variable), sex, education (≥high school vs. <high school), current smoker (yes vs. no), BMI (continuous), hypertension (yes vs. no), diabetes mellitus (yes vs. no), MI or stroke history (yes vs. no), use or nonuse of nephrotoxic medication, rural versus urban resident, TG (continuous), and LDL-C (continuous). The eGFR (continuous) or ACR categories (30-299 mg/g vs. <30 mg/g and ≥300 mg/g vs. <30 mg/g) were also adjusted jointly. Proportional hazards assumptions were verified by testing the interaction with time using the likelihood ratio test, which yielded non-significant *p*-values. We modeled eGFR and ACR using linear spline with knots at 30 mL/min/1.73 m^2^, 45 mL/min/1.73 m^2^, 60 mL/min/1.73 m^2^, and 90 mL/min/1.73 m^2^ for eGFR and 10 mg/g, 30 mg/g, and 300 mg/g for ACR. The interactions between age and eGFR or ACR categories, as well as the two CKD indicators themselves, were tested by including the interaction terms in the model. The joint association between eGFR and ACR with the risk of mortality was also investigated. The risk of mortality was compared for 6 categories of the combinations of eGFR (≥90, 60–89, <60 mL/min/1.73 m^2^) and ACR (<30, ≥30 mg/g) with the category of eGFR ≥ 90 mL/min/1.73 m^2^ and ACR < 30 mg/g as the reference group.

All analyses were conducted with SAS software (version 9.4, SAS institute, CA, USA). The *p*-value of 0.05 was considered statistically significant.

## Results

The mean age of the population at baseline was 49.6 ± 15.2 years, and 42.7% of the participants were male. The mean eGFR was 92.0 ± 18.7 mL/min per 1.73m^2^ and the median level of ACR was 6.6 (IQR: 3.1, 13.6) mg/g. Compared to those without reduced kidney function, participants with eGFR < 60 mL/min per 1.73m^2^ were older, with a higher percentage of nephrotoxic medication users, history of MI or stroke, hypertension and diabetes, lower percentage of male, high-school education or above, and current smokers, as well as higher levels of BMI, TG, and LDL-C. Similar patterns were observed between participants with ACR of ≥30 mg/g and those with <30 mg/g. However, the difference for each risk factor was smaller between people with and without kidney damage (Table 1).

**Table 1:** Baseline characteristics of participants by indicators of CKD

Characteristics	Total(*n* = 47,204)	eGFR < 60 mL/min per 1.73 m^2^(*n* = 2090)	eGFR ≥ 60 mL/min per 1.73 m^2^(*n* = 45,114)	ACR ≥ 30 mg/g(*n* = 5472)	ACR < 30 mg/g(*n* = 41,732)
Age (years)	49.6 ± 15.2	67.2 ± 13.3	48.8 ± 14.8	52.8 ± 15.6	49.2 ± 15.1
Men	20,148(42.7%)	665(31.8%)	19,483(43.2%)	2117(38.7%)	18,031(43.2%)
Rural residents	21,859(46.3%)	950(45.5%)	20,909(46.3%)	2338(42.7%)	19,521(46.8%)
Educated to high school or above^a^	20,950(44.5%)	515(24.7%)	20,435(45.4%)	2183(40.0%)	18,767(45.1%)
Current smoker	11,094(23.5%)	350(16.8%)	10,744(23.8%)	1136(20.8%)	9958(23.9%)
Nephrotoxic medication used	1536(3.3%)	119(5.7%)	1417(3.1%)	236(4.3%)	1300(3.1%)
History of MI or stroke^a^	1220(2.9%)	181(10.0%)	1039(2.6%)	204(4.0%)	1016(2.7%)
Hypertension^a^	16,691(35.4%)	1275(61.1%)	15,329(34.2%)	2655(48.6%)	13,949(33.6%)
Diabetes^a^	3488(7.4%)	341(16.3%)	3147(7.0%)	736(13.5%)	2752(6.6%)
Body-mass index (kg/m^2^) ^a^	23.9 ± 3.7	24.3 ± 3.8	23.9 ± 3.7	24.4 ± 3.9	23.8 ± 3.7
Triglycerides (mmol/L)^a^	1.1(0.80, 1.7)	1.4(0.97, 2.0)	1.1(0.76, 1.7)	1.3(0.87, 1.9)	1.1(0.76, 1.7)
LDL cholesterol (mmol/L)^a^	2.9 ± 0.95	3.2 ± 1.1	2.9 ± 0.94	2.9 ± 0.93	3.0 ± 0.95
Creatinine (μmol/L)	74.8 ± 20.6	113.8 ± 53.4	73.0 ± 15.4	80.6 ± 28.6	74.0 ± 19.1
eGFR (mL/min per 1.73m^2^)	92.0 ± 18.7	51.1 ± 9.4	93.9 ± 16.7	84.1 ± 19.3	93.1 ± 18.4
ACR (mg/g creatinine; median[IQR])	6.6(3.1, 13.6)	10.2(3.9, 30.8)	6.6(3.1, 13.3)	74.5(42.7, 156.0)	5.8(2.6, 10.2)

*Abbreviations CKD* chronic kidney disease, *eGFR* estimated glomerular filtration rate, *ACR* albumin creatinine ratio, *MI* myocardial infarction, *LDL* low density lipoprotein

Data are n(%) or mean ± SD, unless stated otherwise. ACR presented as median and interquartile range because of high skew. ^a^Missing counts: 119 – education level: 4870 – cardiovascular disease history; 243 – hypertension status; 45 – diabetes mellitus: 333 – body-mass index; 41 – triglycerides; 5559 – LDL cholesterol

**Fig. 1:**
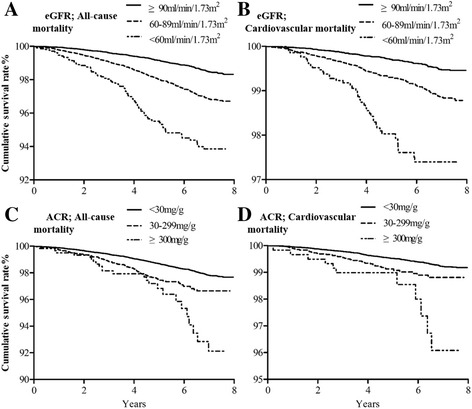
Survival rates according to different levels of estimated glomerular filtration rate (eGFR) and albumin-to-creatinine ratio (ACR). **a** Survival rates for all-cause mortality according to eGFR categories. **b** Survival rates for cardiovascular mortality according to eGFR categories. **c** Survival rates for all-cause mortality according to ACR categories. (**d**) Survival rates for cardiovascular mortality according to ACR categories

The median follow-up duration was 6.1 (IQR: 4.7, 7.5) years. During follow-up, 878 deaths occurred, and 35.6% of them (*n* = 313) were cardiovascular mortality. The overall and cardiovascular mortality rates were 3.2 and 1.1 per 1000 person-years, respectively. Compared to those without indicators of CKD, participants with advanced eGFR or ACR levels had a higher mortality rate (all *p*-values of log-rank test <0.001 for comparisons among eGFR or ACR categories) (Table 2 and Figure 1).

**Table 2:** Crude death rates by CKD indicators

Indicators of CKD		Number	Number of events	Events per 1000 person-years	*P-value* for log-rank test
All-cause mortality					
eGFR	≥90 mL/min/1.73 m^2^	26,169	312	2.0	<0.001
	60–89 mL/min/1.73 m^2^	18,945	466	4.3	
	<60 mL/min/1.73 m^2^	2090	100	8.9	
ACR	<30 mg/g	41,732	725	3.0	<0.001
	30-299 mg/g	4874	125	5.0	
	≥300 mg/g	598	28	9.4	
Total		47,204	878	3.2	NA
Cardiovascular mortality					
eGFR	≥90 mL/min/1.73 m^2^	26,169	102	0.7	<0.001
	60–89 mL/min/1.73 m^2^	18,945	168	1.6	
	<60 mL/min/1.73 m^2^	2090	43	3.8	
ACR	<30 mg/g	41,732	257	1.0	<0.001
	30-299 mg/g	4874	43	1.7	
	≥300 mg/g	598	13	4.4	
Total		47,204	313	1.1	NA

*Abbreviations CKD* chronic kidney disease, *eGFR* estimated glomerular filtration rate, *ACR* albumin creatinine ratio

**Fig. 2:**
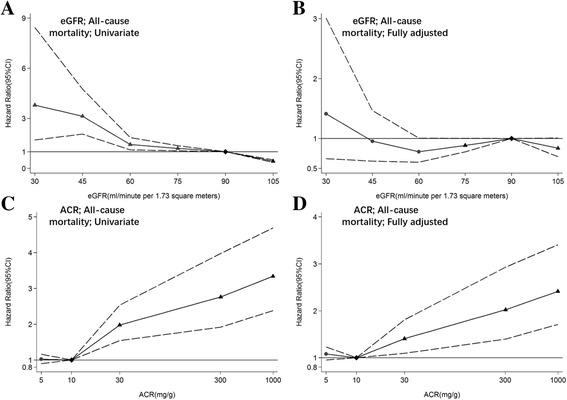
Hazard ratios and 95% CIs for all-cause mortality according to spline estimated glomerular filtration rate (eGFR) and albumin-to-creatinine ratio (ACR). **a**, **b** Hazard ratios and 95% CIs (area between dash lines) in univariable and multivariable adjusted analysis according to eGFR. The reference (diamond) was eGFR 90 mL/min/1.73 m^2^. **c**, **d** Hazard ratios and 95% CIs (area between dash lines) in univariable and multivariable adjusted analysis according to ACR. The reference (diamond) was ACR 10 mg/g. Triangles represent statistically significant and circles represent not significant

**Fig. 3:**
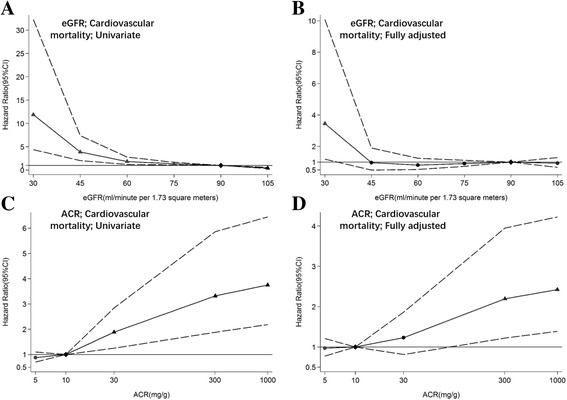
Hazard ratios and 95% CIs for cardiovascular mortality according to spline estimated glomerular filtration rate (eGFR) and albumin-to-creatinine ratio (ACR). **a**, **b** Hazard ratios and 95% CIs (area between dash lines) in univariable and multivariable adjusted analysis according to eGFR. The reference (diamond) was eGFR 90 mL/min/1.73 m^2^. **c**, **d** Hazard ratios and 95% CIs (area between dash lines) in univariable and multivariable adjusted analysis according to ACR. The reference (diamond) was ACR 10 mg/g. Triangles represent statistically significant and circles represent not significant

Compared to those with eGFR ≥ 90 mL/min/1.73 m^2^, participants with eGFR of 60–89 and <60 mL/min/1.73 m^2^ was associated with increased risk of all-cause mortality, with HRs of 2.17 (95% CI: 1.88–2.50) and 4.50 (95% CI: 3.59–5.64), respectively, in the univariable analysis. However, when age and sex were adjusted, the significant associations disappeared. A similar pattern of association was observed between advanced eGFR categories and cardiovascular mortality. Regarding albuminuria, significant associations with all-cause mortality were observed through univariable and multivariable analyses. Albuminuria A3 (≥300 mg/g) was associated with a higher risk of mortality than albuminuria A2 (30–299 mg/g), and compared with albuminuria A1. Similar findings were seen for cardiovascular mortality. However, the associations of albuminuria A2 (30–299 mg/g) with cardiovascular mortality were not significant after adjusting for covariates (Table 3). We modeled eGFR and ACR in linear spline and found a higher risk of all-cause mortality associated with eGFR < 90 mL/min/1.73 m^2^, but the associations were non-significant after adjusting for covariates. Reduced risk of all-cause mortality was associated with eGFR > 90 mL/min/1.73 m^2^ in both univariable and multivariable adjusted analyses. However, for ACR, a significantly higher risk of all-cause mortality can be seen in both univariable and multivariable adjusted analyses, when ACR is greater than 10 mg/g. No significant associations can be found when ACR is less than 10 mg/g (Figure 2). Similar findings can be detected for cardiovascular mortality (Figure 3). Sensitivity analysis by using the eGFR estimated by a modified MDRD equation obtained similar results (data not shown).

**Table 3:** Hazard ratios for all-cause and cardiovascular mortality by indicators of CKD

Indicators of CKD		Univariable model	Multivariable adjusted model 1	Multivariable adjusted model 2	Multivariable adjusted model 3
All-cause mortality					
eGFR	≥90 mL/min/1.73 m^2^	Reference	Reference	Reference	Reference
	60–89 mL/min/1.73 m^2^	2.17(1.88–2.50)	0.98(0.84–1.16)	0.98(0.83–1.15)	0.96(0.82–1.13)
	<60 mL/min/1.73 m^2^	4.50(3.59–5.64)	1.13(0.88–1.47)	1.09(0.84–1.41)	1.03(0.79–1.34)
ACR	<30 mg/g	Reference	Reference	Reference	Reference
	30-299 mg/g	1.74(1.44–2.11)	1.39(1.15–1.69)	1.26(1.04–1.53)	1.26(1.04–1.53)
	≥300 mg/g	3.32(2.28–4.85)	2.29(1.57–3.35)	2.07(1.41–3.04)	2.07(1.40–3.04)
Cardiovascular mortality					
eGFR	≥90 mL/min/1.73 m^2^	Reference	Reference	Reference	Reference
	60–89 mL/min/1.73 m^2^	2.39(1.87–3.06)	0.95(0.72–1.26)	0.91(0.68–1.20)	0.89(0.67–1.18)
	<60 mL/min/1.73 m^2^	5.93(4.15–8.47)	1.20(0.79–1.82)	1.05(0.69–1.59)	0.99(0.65–1.50)
ACR	<30 mg/g	Reference	Reference	Reference	Reference
	30-299 mg/g	1.69(1.22–2.34)	1.30(0.94–1.80)	1.08(0.78–1.50)	1.08(0.77–1.50)
	≥300 mg/g	4.36(2.50–7.61)	2.84(1.63–4.97)	2.36(1.34–4.16)	2.32(1.31–4.12)

*Abbreviations*: *CKD* chronic kidney disease, *eGFR* estimated glomerular filtration rate, *ACR* albumin creatinine ratio, *LDL* low density lipoprotein

Effects of kidney damage markers on mortality were expressed as hazard ratios and 95% confidence intervals. Model 1 was adjusted for age, sex. Model 2 was adjusted for all variables in Model 1 plus education, current smoking, body mass index, hypertension, diabetes mellitus, cardiovascular disease history, use of nephrotoxic medication, rural or urban residents, high triglycerides, high LDL cholesterol. Model 3 was adjusted for all variables in Model 2 plus eGFR or ACR categories, as appropriate

Significant interactions between level of eGFR and age were detected in the associations for both all-cause mortality (*p*-value for interaction < 0.001) and cardiovascular mortality (*p*-value for interaction = 0.02). No interactions were detected between ACR categories and age, and ACR categories and the two CKD indicators. Hence, we initiated association analyses stratified by age groups (≥65 years or <65 years). The associations among the participants aged less than 65 years were more pronounced than in those aged over 65 years. For example, the fully-adjusted HR for all-cause mortality was 1.64 (95% CI: 1.02–2.64) for eGFR <60 mL/min/1.73 m^2^ among people aged less than 65 years, compared to 0.86 (95% CI: 0.61–1.21) for eGFR ≥90 mL/min/1.73m^2^ among people aged more than 65 years (Additional file 1: Tables S1 and S2).

In the joint analysis of eGFR and ACR, we observed a similar pattern of increased mortality rates for ACR ≥ 30 mg/g compared with ACR <30 mg/g across all levels of eGFR and vice versa. However, there were no significant associations of joint categories of eGFR and ACR with both all-cause and cardiovascular mortality (Table 4).

**Table 4:** Joint mortality risk estimation and hazard ratios for mortality according to categories of eGFR and ACR

eGFR (mL/min/1.73 m^2^)	ACR < 30 mg/g				ACR ≥ 30 mg/g			
	Number	Number of events	Events per 1000 person-years	Fully adjusted HR(95%CI)^a^	Number	Number of events	Events per 1000 person-years	Fully adjusted HR(95%CI)^a^
All-cause mortality								
≥90	24,075	283	2.0	Reference	2094	29	2.5	1.02 (0.69–1.49)
60–89	16,104	373	4.0	0.92 (0.77–1.09)	2841	93	6.7	1.39 (1.08–1.79)
<60	1553	69	8.1	1.02 (0.76–1.37)	537	31	11.3	1.36 (0.92–2.02)
Cardiovascular mortality								
≥90	24,075	91	0.6	Reference	2094	11	1.0	1.05 (0.56–1.97)
60–89	16,104	134	1.4	0.86 (0.64–1.16)	2841	34	2.4	1.22 (0.79–1.87)
<60	1553	32	3.8	1.07 (0.68–1.70)	537	11	4.0	1.04 (0.53–2.02)

*Abbreviations eGFR* estimated glomerular filtration rate, *ACR* albumin creatinine ratio, *HR* hazard ratio, *CI* confidence interval

^a^Adjusted for age, sex, education, current smoking, body mass index, hypertension, diabetes mellitus, cardiovascular disease history, use of nephrotoxic medication, rural or urban residents, triglycerides, and low density lipoprotein cholesterol

## Discussion

In our study conducted among a general Chinese population, increased ACR was found to be independently associated with increased risk of both all-cause and cardiovascular mortality. Reduced eGFR was only found to be associated with increased risk of the adverse outcomes in univariable analysis, but the association disappeared after adjusting for age and sex. Furthermore, the associations were modified by age, and seem to be more prominent among participants aged < 65 years than in those aged ≥ 65 years.

An increased level of urinary albumin excretion is an early sign of kidney damage and persistent albuminuria, typically ACR ≥ 30 mg/g lasting for more than three months, has been included as a diagnostic standard for CKD [14]. The association of increased ACR with both all-cause mortality and cardiovascular mortality in the general population has been well described in the analyses of the CKD Prognosis Consortium(CKD-PC) [5]. In the above meta-analysis, ACR greater than 5 mg/g or dipstick urine protein 1+ and above were associated with increased risk of adverse outcomes. However, previous studies of Asian populations did not employ ACR as a measurement for albuminuria [15, 16], except for some smaller studies [17, 18]. There is an individual study from the meta-analysis based on population from Taiwan, where people have the same ethnicity as those in mainland China. Wen et al. reported an increased risk of both all-cause and CVD mortality associated with minimal or overt proteinuria in populations with eGFR ≥ 60 mL/min/1.73 m^2^ (CKD stages 1 and 2), compared with people without CKD [4]. In our study, the quantitative measurement of ACR was used to represent albuminuria, and we found results consistent with the above mentioned studies. Furthermore, we found that the effect of increased ACR on mortality was independent of traditional CVD risk factors and eGFR levels. Regarding the spline analysis, we selected the same knots as the above mentioned CKD-PC meta-analysis, but used 10 mg/g instead of 5 mg/g as the reference point due to the limited sample size in our study. However, we did not replicate the protective effect for mortality associated with ACR of less than 10 mg/g [5]. As the effect of ACR could be partly due to its representativeness of other metabolic disorders, e.g. hypertension and diabetes mellitus, it could be assumed that the significant association found in our study could be a reflection of the pathogenic process due to albuminuria itself, e.g. endothelial dysfunction and systemic inflammation [19, 20]. A previous national survey of CKD in China has reported a much higher prevalence of albuminuria than of reduced eGFR [7]. Hence, it may be important to place more resources in the screening and intervention of albuminuria among the general population.

Previous studies consistently demonstrated an increased risk of adverse outcomes associated with reduced kidney function among the general population [5, 21, 22]. Our study, however, failed to fully replicate the previous findings among a large sample of a general Chinese population, as the significant association between reduced eGFR and adverse outcomes turned out to be null after adjusting for the age of the population. In our study, a small percentage of the population had impaired kidney function (eGFR < 60 mL/min/1.73 m^2^) (*n* = 2090, 4.4%). Furthermore, the present study found a very high percentage of elderly among CKD versus non-CKD patients (62.3% versus 14.7%). Therefore, the non-significant association between reduced eGFR and mortality may be due to the limited number of people with exposure and a significant confounding effect of age.

Furthermore, we found that, aside from being an important confounding factor, age is also an effect modifier for eGFR. Several studies have shown that the effect of kidney insufficiency on the occurrence of cardiovascular disease and death is modified by age. For example, Raymond et al. used the central laboratory system data of 106,366 participants in Coventry, UK, and found that the relative risk of mortality in a population of 60 years of age and above was increasing more slowly than that in the younger population and the value of relative risk was also smaller among elderly adults than younger adults [23]. A US study conducted among veterans suggested the association of mortality risk with impaired kidney function was attenuated in people older than 75 years [24]. However, based on a large-scale study of a Norwegian general population, Hallan et al. found that the association between reduced kidney function and cardiovascular mortality tended to be stronger in participants aged over 70 years than in those aged less than 70 years [25]. Our study supports the former finding that reduced eGFR exerts a more obvious impact on mortality among a younger population than among an older population. The reason for the interaction may be due to the high mortality rate and high prevalence of co-morbidities of CKD (e.g. hypertension and diabetes) among the elderly, which could make the effect of reduced eGFR alone less significant.

Although our study has the advantage of a large sample size of a general population, as well as longitudinal validation of adverse outcomes, some limitations should be noted. First, biomarkers including eGFR and ACR were measured in different study centers. Although measurement was calibrated with the standard sample from the central laboratory and under tight quality control, some variation among different centers might still exist. Second, the two race CKD-EPI equation was not developed for Asian populations and may limit its accuracy for estimating GFR in the Chinese population. Nevertheless, it might perform as well as the modified MDRD equation for the Chinese population, as shown by an evaluation study among 977 adult Chinese individuals [26]. Third, given the likely under-reporting rate of death, the mortality rate among different groups of participants in our study might be underestimated. Fourth, the number of study participants in late CKD stages was limited, with less than 1% of the total population in stages 3B to 5. Only 11.6% of the population had ACR > 30 mg/g. As a result, there was not enough study power to evaluate the full effect for all five stages of CKD. Fifth, we used baseline status for analyses, and subsequent measurements for changes in risk factors were not conducted. At the same time, albuminuria and reduced kidney function determined by a single measurement did not conform to the ≥3 month persistence criteria to diagnose CKD.

## Conclusions

Our study was a further attempt to study a large Asian population to determine the relationship between albuminuria and mortality. However, the association of reduced kidney function with mortality was not clear.

## Additional file

Additional file 1: Table S1. and **S2.**Hazard ratios for all-cause and cardiovascular mortality by indicators of chronic kidney disease among participants aged less than 65 years old, as well as among those aged more than 65 years old. (DOCX 17 kb)

## Abbreviations

ACR: Albumin creatinine ratio
BMI: Body-mass index
BP: Blood pressure
CI: Confidence interval
CKD: Chronic kidney disease
CVD: Cardiovascular disease
eGFR: estimated glomerular filtration rate
HR: Hazard ratios
IQR: Interquartile range
LDL-C: Low density lipoprotein cholesterol
MI: Myocardial infarction
SD: Standard deviation
TG: Serum triglycerides
