# Supplementary material for: Reduced Kidney Function, Albuminuria, and Risks for All-cause and Cardiovascular Mortality in China: A Population-based Cohort Study
Source: BMC Nephrol. 2017 Jun 7;18:188. doi: 10.1186/s12882-017-0603-9 (PMC5463353; doi:10.1186/s12882-017-0603-9)
Supplement: Additional file 1: Table S1. — and S2.Hazard ratios for all-cause and cardiovascular mortality by indicators of chronic kidney disease among participants aged less than 65 years old, as well as among those aged more than 65 years old. (DOCX 17 kb) [file 12882_2017_603_MOESM1_ESM.docx]

| **Supplemental table 1.** Hazard ratios for all-cause and cardiovascluar mortality by indicators of CKD among participants aged less than 65 years old | | | | | |
| --- | --- | --- | --- | --- | --- |
| Indicators of kidney damage | | Univariate model | Multi-variate adjusted model1 | Multi-variate adjusted model2 | Multi-variate adjusted model3 |
| All cause mortality | | | | | |
| eGFR | ≥90 ml/min/1.73m^2^ | Reference | Reference | Reference | Reference |
|  | 60-89 ml/min/1.73m^2^ | 1.63(1.34-1.97) | 1.11(0.91-1.35) | 1.09(0.9-1.33) | 1.08(0.89-1.32) |
|  | <60 ml/min/1.73m^2^ | 3.30(2.09-5.20) | 1.97(1.24-3.13) | 1.80(1.13-2.86) | 1.64(1.02-2.64) |
| ACR | <30mg/g | Reference | Reference | Reference | Reference |
|  | 30-299mg/g | 1.51(1.13-2.03) | 1.43(1.07-1.92) | 1.28(0.95-1.72) | 1.25(0.93-1.69) |
|  | ≥300mg/g | 3.14(1.72-5.71) | 2.77(1.52-5.05) | 2.32(1.27-4.26) | 2.11(1.14-3.91) |
| Cardiovascular mortality | | | | | |
| eGFR | ≥90 ml/min/1.73m^2^ | Reference | Reference | Reference | Reference |
|  | 60-89 ml/min/1.73m^2^ | 1.54(1.09-2.17) | 1.01(0.71-1.44) | 0.95(0.67-1.36) | 0.95(0.66-1.35) |
|  | <60 ml/min/1.73m^2^ | 4.17(2.01-8.64) | 2.44(1.16-5.13) | 1.86(0.88-3.96) | 1.70(0.78-3.72) |
| ACR | <30mg/g | Reference | Reference | Reference | Reference |
|  | 30-299mg/g | 1.34(0.77-2.34) | 1.27(0.73-2.22) | 1.04(0.59-1.81) | 1.01(0.58-1.78) |
|  | ≥300mg/g | 3.64(1.35-9.87) | 3.23(1.19-8.76) | 2.20(0.80-6.04) | 1.94(0.69-5.43) |
| *Note:* Effects of kidney damage markers on mortality were expressed as hazard ratios and 95% confidence intervals. Model1 was adjusted for age, sex. Model2 was adjusted variables in model1 plus education, current smoking, body mass index, hypertension, diabetes mellitus, cardiovascular disease history, use of nephrotoxic medication, rural or urban residents, high triglyceride, high low density lipoprotein cholesterol. Model3 was adjusted variables in model2 plus eGFR or ACR categories in appropriate. Abbreviations: CKD, chronic kidney disease; eGFR, estimated glomerular filtration rate; ACR, albumin creatinine ratio. | | | | | |

| **Supplemental table 2.** Hazard ratios for all-cause and cardiovascular mortality by indicators of CKD among participants aged more than 65 years old | | | | | |
| --- | --- | --- | --- | --- | --- |
| Indicators of kidney damage | | Univariate model | Multi-variate adjusted model1 | Multi-variate adjusted model2 | Multi-variate adjusted model3 |
| All cause mortality | | | | | |
| eGFR | ≥90 ml/min/1.73m^2^ | Reference | Reference | Reference | Reference |
|  | 60-89 ml/min/1.73m^2^ | 0.90(0.70-1.15) | 0.75(0.58-0.98) | 0.80(0.61-1.04) | 0.79(0.60-1.03) |
|  | <60 ml/min/1.73m^2^ | 1.15(0.84-1.57) | 0.86(0.62-1.21) | 0.90(0.64-1.27) | 0.86(0.61-1.21) |
| ACR | <30mg/g | Reference | Reference | Reference | Reference |
|  | 30-299mg/g | 1.40(1.09-1.80) | 1.37(1.07-1.77) | 1.24(0.96-1.61) | 1.26(0.98-1.63) |
|  | ≥300mg/g | 2.12(1.30-3.45) | 2.05(1.26-3.35) | 1.93(1.18-3.17) | 2.07(1.25-3.42) |
| Cardiovascular mortality | | | | | |
| eGFR | ≥90 ml/min/1.73m^2^ | Reference | Reference | Reference | Reference |
|  | 60-89 ml/min/1.73m^2^ | 1.04(0.69-1.59) | 0.80(0.51-1.24) | 0.83(0.53-1.29) | 0.81(0.52-1.27) |
|  | <60 ml/min/1.73m^2^ | 1.45(0.88-2.39) | 0.92(0.53-1.58) | 0.91(0.53-1.56) | 0.85(0.49-1.47) |
| ACR | <30mg/g | Reference | Reference | Reference | Reference |
|  | 30-299mg/g | 1.37(0.92-2.05) | 1.30(0.87-1.95) | 1.11(0.74-1.68) | 1.12(0.74-1.69) |
|  | ≥300mg/g | 2.83(1.44-5.57) | 2.67(1.36-5.24) | 2.43(1.22-4.83) | 2.53(1.26-5.08) |
| *Note:* Effects of kidney damage markers on mortality were expressed as hazard ratios and 95% confidence intervals. Model1 was adjusted for age, sex. Model2 was adjusted variables in model1 plus education, current smoking, body mass index, hypertension, diabetes mellitus, cardiovascular disease history, use of nephrotoxic medication, rural or urban residents, high triglyceride, high low density lipoprotein cholesterol. Model3 was adjusted variables in model2 plus eGFR or ACR categories in appropriate. Abbreviations: CKD, chronic kidney disease; eGFR, estimated glomerular filtration rate; ACR, albumin creatinine ratio. | | | | | |
